# Low patient activation levels in frail older adults: a cross-sectional study

**DOI:** 10.1186/s12877-017-0696-9

**Published:** 2018-01-05

**Authors:** Anouk Overbeek, Judith A. C. Rietjens, Lea J. Jabbarian, Johan Severijnen, Siebe J. Swart, Agnes van der Heide, Ida J. Korfage

**Affiliations:** 1 Department of Public Health, Erasmus MC, PO Box 2040, 3000 CA Rotterdam, the Netherlands; 2 Laurens, Rotterdam, the Netherlands; 3 Swinhove Groep, Zwijndrecht, the Netherlands

**Keywords:** Patient activation, Patient activation measure, Older adults, Health-related quality of life, Frailty

## Abstract

**Background:**

Frail older adults are increasingly expected to self-manage their health and healthcare. We assessed the extent to which this group is able to take up this responsibility by measuring their level of activation as patients (i.e. their knowledge, skills and confidence to self-manage their health and healthcare). Further, we studied which characteristics of older adults were associated with patient activation.

**Methods:**

In this cross-sectional study 200 frail, competent adults (median age 87 years) participated. Participants were community-dwelling adults who received home care and residents of care homes. Data were collected via personal interviews in participants’ homes**.** The main outcome measure was patient activation assessed by the short version of the Patient Activation Measure (PAM-13; range: 0–100). The PAM distinguishes four levels of increasing activation with level 1 indicating poor patient activation and level 4 adequate patient activation. Other studied variables were: multimorbidity, type of residency, frailty (Tilburg Frailty Index), mental competence (Mini Mental State Examination), health-related quality of life (SF-12), satisfaction with healthcare (subscale Patient Satisfaction Questionnaire) and personal characteristics (age, gender, marital status, educational level). Regression analyses were performed to investigate which variables were associated with patient activation.

**Results:**

Participants had a median PAM score of 51. Thirty-nine percent had level 1 activation, 31% level 2, 26% level 3 and 5% level 4. Fifty-nine percent of community dwelling adults had level 1 or 2 activation versus 81% of care home residents (*p* = 0.007). Mental competence (Effect: 0.52, CI: 0.03–1.01, *p* = 0.04) and health-related quality of life (Effect: 0.15, CI: 0.01–0.30, *p* = 0.04 for physical health; Effect: 0.20, CI: 0.07–0.34, *p* = 0.003 for mental health) were positively associated with patient activation. Frailty (Effect: -1.06, CI: -1.75 – -0.36, *p* = 0.003) was negatively associated with patient activation.

**Conclusions:**

The majority of this frail and very old study population, especially those with a lower health-related quality of life, may be unable to self-manage their health and healthcare to the level expected from them. The increasing population of frail older adults may need help in managing their health and healthcare.

## Background

“Frailty is a process of an accumulation of physical, psychological and/or social deficits in functioning which increase the chance of adverse health outcomes (functional disabilities, admission to an institution, death) [1].” In the Netherlands, around 27% of older adults aged 65 and over are frail. This percentage includes older adults living independently (of whom one quarter are frail) and older people living in an institution, e.g. a care home (of whom three-quarters are frail) [1].

Currently, governments in many Western countries including the Netherlands are reforming their healthcare sectors [2] and develop policies that aim to reduce institutional care, thereby encouraging frail older adults to remain community-dwelling [3, 4]. Since these efforts align with the preferences of many older adults [5], these policies may well be successful. However, their success relies on the degree of older adults’ ability to manage their lives, health and healthcare by themselves while community-dwelling [6], where possible assisted by (informal) caregivers. The question is whether frail older adults have sufficient abilities to do so. Older adults often have lower health literacy skills than younger adults [7] and often find it difficult to take an active role in healthcare decision-making [8]. This suggests that the levels of patient activation in older adults may be relatively low.

Patient activation refers to the motivation, knowledge, skills and confidence that equip adults to be actively engaged in their health and healthcare [9]. Hibbard et al. came up with the following conceptual definition of patient activation: “Those who are activated believe patients have important roles to play in self-managing care, collaborating with providers, and maintaining their health. They know how to manage their condition and maintain functioning and prevent health declines; and they have the skills and behavioral repertoire to manage their condition, collaborate with their health providers, maintain their health functioning, and access appropriate and high-quality care [9].” According to Hibbard et al., activation is developmental in nature and involves four increasing levels [9]. Cross-sectional studies among populations with a range of conditions and economic backgrounds reported that adults’ activation levels were positively related to their health status (e.g. health-related quality of life, which refers to the perceived well-being in physical, mental and social domains of life), healthy behaviors (e.g. physical activity, healthy diet), appropriate use of healthcare systems (e.g. not delaying doctor visits) and satisfaction with care services [10, 11]. Females, younger adults and those with higher education and income levels have been shown to have higher activation levels [12, 13]. Longitudinal studies suggest that the level of patient activation is also predictive of future health outcomes [14, 15]. For instance, Hibbard et al. assessed activation levels of adults with chronic conditions [15]. After four years of follow-up, less activated adults had “significant worse levels of medication adherence, getting recommended care, health behaviors, functional health, emergency department use, and hospitalizations” than the most activated adults [15].

Until now, only few studies have investigated patient activation among older adults [16–19]. The studied adults were not necessarily frail and their mean ages varied from 71 [17] to 77 [18]. Investigating patient activation among frail, very old adults is relevant because this population has high needs for healthcare and is more and more expected to manage their lives, health and healthcare by themselves. In this explorative study we determined the degree of patient activation in frail, very old adults. Furthermore, we aim to assess which personal (age, gender, marital status and education) and other characteristics (multimorbidity, type of residency, frailty level, mental competence, physical health, mental health and satisfaction with healthcare) are associated with patient activation in this population.

## Methods

### Study design and population

This study had a cross-sectional design. We used data from the baseline assessment in a cluster randomised controlled trial on the effects of Advance Care Planning. Information on the study protocol of the trial can be found elsewhere [20]. The study population consisted of both community-dwelling older adults who received regular care from a home care organisation, such as assistance with activities of daily living (ADL), self-care or domestic help, and older adults residing in one of 16 residential care homes of a large long term care organisation in Rotterdam, the Netherlands. In the Netherlands, adults are admitted to residential care homes if they are no longer able to live on their own home due to illness, disabilities and/or old age. They can get assistance with ADL, self-care, medication use and/or domestic help 24 h a day. However, the medical care and treatment provided by residential care homes is very limited and residents are required to manage this by themselves (e.g. by consulting their general practitioner or other physicians). Dutch nursing homes on the other hand, employ their own medical, paramedical and psychosocial staff, including a specially trained nursing home physician. Their residents are often more disabled and need more help with ADL than residential care home residents [21].

To be eligible for participation, adults had to be ≥75 years, frail and mentally competent. Frailty was operationalised as a score of ≥5 on the Tilburg Frailty Index (TFI, range 0–15) [22]. The TFI consists of a physical (range: 0–8), psychological (range: 0–4) and a social domain (range: 0–3). Mental competency was based on the score of the Mini Mental State Examination (MMSE, range 0–30) [23]. MMSE scores between 0 and 16 are indicative of mental incapacity [24], therefore we used a score of ≥17 as criterion for inclusion. The care staff initially indicated which adults were likely eligible. Eligibility was subsequently confirmed by the research team using the instruments described above.

### Measures

Personal interviews were conducted during the period March 2014 – April 2015. Through a subscale of the TFI, we collected socio-demographic data on age, gender, marital status, multimorbidity (2 diseases or chronic disorders) and education level. Education level was defined as the highest educational qualification achieved (low = none or primary education; middle = secondary education; high = higher professional or university education). To determine the degree of knowledge, skills and confidence for self-management of health and healthcare, we used the short version of the Patient Activation Measure (PAM-13) [9, 13]. The PAM consists of 13 statements, such as “I know how to prevent problems with my health” and “I am confident that I can tell a doctor my concerns, even when he or she does not ask”. The four answer options range from “disagree strongly” to “agree strongly” and a fifth response option is “not applicable”. We used a conversion table provided by the developers (Insignia Health) to calculate a standardised activation score ranging from 0 to 100 (the PAM score). Besides the PAM score, with higher scores indicating more activation, the conversion table automatically calculates four levels of patient activation. At level one (PAM scores ≤47.0), adults “tend to be overwhelmed with the task of managing their health and may not feel ready to take an active role”. At level two (PAM scores between ≥47.1 and ≤55.1), adults “realise that they have a role to play in their healthcare, but may lack the knowledge and confidence to manage their health and healthcare”. At level three (PAM scores between ≥55.2 and ≤72.4), adults “are beginning to take action, but may still lack some confidence to manage all aspects of their health”. At level 4 (PAM scores ≥72.5), adults can manage their health and care, but “struggle with being able to maintain the behaviours they have already adopted” [25]. The PAM is a reliable and valid measure with good psychometric properties [9, 13] and has been shown to be valid in a study of multimorbid older adults with a mean age of 77 [18].

Further, we added outcome variables to the questionnaire that were found relevant for patient activation in previous research [10, 11]. Generic health-related quality of life was measured with the 12-Item Short Form Survey (SF-12) [26] which generates a physical component score (PCS) and a mental component score (MCS, range: 0–100). SF-12 scores of our frail and old study population were compared with the SF-12 scores of the general population aged ≥75 years, using data of “Statistics Netherlands” [27]. General satisfaction with healthcare was measured by one subscale (2 items) of the Patient Satisfaction Questionnaire (PSQ-18; range: 1–5) [28].

### Statistical analysis

Since the scores of the TFI and MMSE were not normally distributed, we decided to report the median and interquartile range (IQR) for all continuous variables including the PAM score, which was normally distributed. A chi-squared test investigated the association between type of residency and PAM levels. Further, we investigated associations between age, gender, marital status, education, multimorbidity, type of residency, frailty level, mental competence, physical health, mental health and satisfaction with healthcare on the one hand, and the PAM score on the other hand using univariate linear regression analysis. To assess whether each of our continuous variables was linearly related to the PAM score, we added a quadratic term of the continuous variable to each model. If continuous variables appeared to be non-linearly associated with the outcome we used a spline function to assess the association between that variable and the outcome (PAM). A multiple linear regression analysis was performed to investigate which variables were associated with the PAM score while controlling for personal characteristics (age, gender, marital status, education). In this multiple regression analysis, we only included variables that were significantly associated with patient activation in univariate analyses. We controlled the residual plot for each included variable. Analyses were performed using IBM SPSS statistics V.22 and using R.

### Ethics

The independent Ethics committee of Rotterdam (Medisch Ethische Toetsingscommissie) proved approval for the study to be performed. Potential candidates received a letter with study information and had the possibility to ask questions in the first personal interview. If candidates were willing to participate and eligible, a second personal interview was arranged. During this interview, written informed consent was obtained.

## Results

**Fig. 1:**
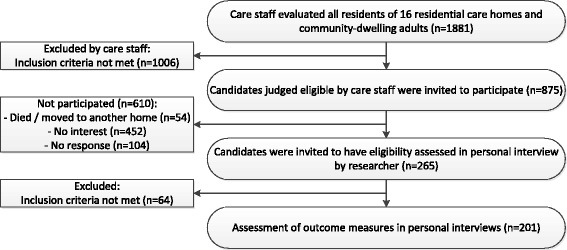
Flow chart

Care staff evaluated 1881 individuals for possible participation in total, of whom 1006 were excluded because they did not fulfil the inclusion criteria. We approached the remaining 875 individuals. 610 out of 875 potential candidates did not participate (54 died or moved to another home, 452 indicated they were not interested and 104 did not respond). The remaining 265 candidates were willing to participate, of whom 201 were indeed eligible and participated (Fig. 1).

**Fig. 2:**
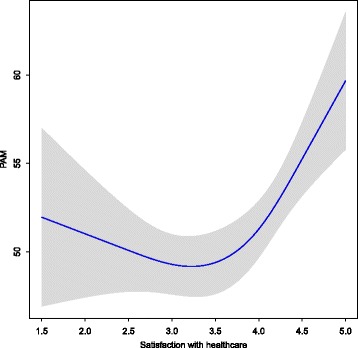
Spline plot of the association of satisfaction with healthcare (x-axis) with the score on the Patient Activation Measure (PAM, y-axis)

Data of one person were excluded due to responding “not applicable” too often (≥4 times) when completing the PAM. The median and mean age of participants was 87 years (Table 1). We found a median of 31 for the physical health (mean score = 32, compared to a mean score of 41 for the general older population [27]) and a median of 51 for the mental health (mean score = 51, compared to a mean score of 53 for the general older population [27]). Since the quadratic term of the PSQ score (subscale “General Satisfaction”) was significant, indicating a non-linear association between the PSQ score and the PAM score, we calculated a spline function (Fig. 2), which showed a significant relationship of the PSQ and the PAM.

**Table 1:** Characteristics of the study population

Sample characteristic	Sample description (*n* = 200)
Age, median (IQR, range)	87 (7.8, 73–102)
Gender, *n* (%)	
- female	140 (70)
Marital status, *n* (%)	
- married / cohabiting	39 (20)
- never married	16 (8)
- divorced	10 (5)
- widow(er)	135 (68)
Education level, *n* (%)	
- high	21 (11)
- middle	104 (52)
- low	74 (37)
*- missing*	*1*
Multimorbidity, *n* (%)	
- yes	95 (48)
-*missing*	*1*
Type of residency, *n* (%)	
- community-dwelling	110 (55)
- care home	90 (45)
*Frailty*	
Tilburg Frailty Index (TFI), median (IQR, range)^a^	7 (3.0, 5-14)
- physical domain, median (IQR, range)^b^	5 (2.0, 1-8)
- psychological domain, median (IQR, range)^c^	1 (1.0, 0-4)
- social domain, median (IQR, range)^d^	2 (1.0, 0-3)
*Competence*	
Mini Mental State Examination (MMSE), median (IQR, range)^e^	27 (4.0, 20-30)
*Patient activation*	
Patient Activation Measure (PAM), median (IQR, range)^f^	51 (10.3, 33-100)
Activation levels based on PAM score, *n* (%)^g^	
- level 1 (≤47.0)	77 (39)
- level 2 (≥47.1 and ≤ 55.1)	61 (31)
- level 3 (≥55.2 and ≤72.4)	52 (26)
- level 4 (≥72.5)	10 (5)
*Generic health-related quality of life*	
SF-12	
- physical health component score (PCS-12), median (IQR, range)^h^	31 (11.9, 10-68)
- mental health component score (MCS-12), median (IQR, range)^h^	51 (13.1, 22-75)
*Satisfaction with healthcare*	
Subscale “General Satisfaction” of the Patient Satisfaction Questionnaire (PSQ-18), median (IQR, range)^i^	4 (1.0, 1.5-5)
- score 1.00- 2.00, *n* (%)	14 (7)
- score 2.50- 3.50, *n* (%)	79 (40)
- score 4.00- 5.00, *n* (%)	107 (54)

^a^TFI, normal range 0–15. Higher scores indicate worse functioning. ^b^ TFI physical domain, normal range 0–8. ^c^ TFI psychological domain, normal range 0–4. ^d^ TFI social domain, normal range 0–3. ^e^ MMSE, normal range 0–30. Higher scores indicate better functioning. ^f^ PAM, normal range 0–100. Higher scores indicate a higher degree of engagement in health behavior. ^g^ Higher levels indicate a higher degree of engagement in health behavior. ^h^ SF-12, normal range 0–100. Higher scores indicate better functioning. ^i^ Subscale PSQ-18, normal range 1–5. Higher scores indicate better functioning

### Patient activation

The median PAM score was 51 (IQR: 10.3, range: 33–100). Most adults reported activation level 1 (39%), followed by activation level 2 (31%), activation level 3 (26%) and activation level 4 (5%; Table 1). Care home residents more often reported lower activation levels (levels 1 and 2; *n* = 73, 81%) than community-dwelling adults (*n* = 65, 59%, *p* = 0.007, Table 2).

**Table 2:** Activation levels by type of residency

Type of residency	Activation levels, *n* (%)			
	Level 1	Level 2	Level 3	Level 4
Community dwelling, *n* = 110	37 (34)	28 (26)	39 (36)	6 (6)
Care home, *n* = 90	40 (44)	33 (37)	13 (14)	4 (4)

Being community dwelling (compared to care home residents; Effect: 2.74, Cl: 0.12–5.36, *p* = 0.04), the degree of mental capacity (Effect: 0.57, Cl: 0.06–1.07, *p* = 0.03), physical health (Effect: 0.20, Cl: 0.07–0.33, *p* = 0.004) and mental health (Effect: 0.27, Cl: 0.15–0.40, *p* < 0.001) and satisfaction with healthcare (Effect: 1.99, CI: 0.37–3.62, *p* < 0.001) were positively associated with PAM scores. Education level (Effect: −4.74, Cl: −9.28 – −0.21 for high educated adults compared to low educated adults, *p* = 0.05) and frailty (Effect: −1.64, Cl: −2.24 – −1.04, *p* < 0.001) were negatively associated with PAM scores (Table 3).

**Table 3:** Univariate linear regression between personal and other characteristics of older adults, and patient activation

	Patient Activation Measure (PAM), *n* = 200	
	Effect (95% CI)	*p*-value
Age (per year)	-0.05 (−0.28–0.19)	.71
Gender		.97
- female	-0.05 (-2.92 – 2.82)	
- male	Ref	
Marital status		.85
- married / cohabiting	1.35 (-2.02 – 4.73)	
- never married	-0.01 (-4.92 – 4.90)	
- divorced	-0.90 (-6.98 – 5.19)	
- widow(er)	Ref	
Education level^a^		.05
- high	-4.74 (-9.28 – -0.21)	
- middle	-2.93 (-5.72 – -0.14)	
- low	Ref	
Multimorbidity^a^		
- yes	2.14 (-0.49 – 4.77)	.11
- no	Ref	
Type of residency		.04
- community dwelling	2.74 (0.12 – 5.36)	
- care home	Ref	
*Frailty*		<.001
Tilburg Frailty Index (TFI, per point)	-1.64 (-2.24 – -1.04)	
*Competence*		.03
Mini Mental State Examination (MMSE, per point)	0.57 (0.06 – 1.07)	
Generic health-related quality of life		
SF-12		
- physical health component score (PCS-12, per point)	0.20 (0.07 – 0.33)	.004
- mental health component score (MCS-12, per point)	0.27 (0.15 – 0.40)	<.001
*Satisfaction with healthcare*		
Subscale “General Satisfaction” of the Patient Satisfaction Questionnaire (PSQ-18), interquartile distance (4 vs. 3)	1.99 (0.37 – 3.62)	<.001

^a^ *Missing = 1*

Multiple regression analysis (Table 4) confirmed these findings, however, the association between satisfaction with healthcare and PAM scores was no longer significant.

**Table 4:** Multiple linear regression between characteristics of older adults and patient activation^a^

	Patient Activation Measure (PAM), *n* = 199	
	Effect (95% CI)	*p*-value
Type of residency		.009
- community dwelling	3.49 (0.89 – 6.09)	
- care home	Ref	
*Frailty*		.003
Tilburg Frailty Index (TFI, per point)	-1.06 (-1.75 – -0.36)	
*Competence*		.04
Mini Mental State Examination (MMSE, per point)	0.52 (0.03 – 1.01)	
*Generic health-related quality of life*		
SF-12		
- physical health component score (PCS-12, per point)	0.15 (0.01 – 0.30)	.04
- mental health component score (MCS-12, per point)	0.20 (0.07 – 0.34)	.003
*Satisfaction with healthcare*		.06
Subscale “General Satisfaction” of the Patient Satisfaction Questionnaire (PSQ-18), interquartile distance (4 vs. 3)	0.84 (-0.71 – 2.40)	

^a^ *The model is adjusted for age, gender, marital status and education. Adjusted R squared = 0.237*

## Discussion

This study describes a frail, very old population with lower levels of physical functioning than the general older population [27]. A majority of frail older adults in our study had low activation levels (levels 1 and 2 of the PAM). Low activation levels were in particular present among those with a lower health-related quality of life and among care home residents. However, more than half of community dwelling adults had suboptimal activation levels (levels 1 and 2) as well.

Compared to other study populations, such as younger adults with chronic physical disorders (mean PAM scores: 57 [14] - 69 [29] or community-dwelling older adults with lower mean ages (77, mean PAM score: 57 [18] and 74, mean PAM score: 66 [17]), the mean activation score of our participants (mean PAM score: 52) was rather low. This could be related to our participants’ advanced age.

In accordance with previous findings [10, 11], health was positively associated with patient activation in our study. Unlike earlier studies, we found no significant association between age and patient activation [12, 13], which may not be surprising given the homogeneous age of our study population (≥73 years). Unexpectedly, we found higher activation levels among those with low versus high education levels in univariate analyses. This finding could partly be due to a selection effect. In the Netherlands, life expectancy of adults with lower educational levels is 76.6 years for men and 80.2 years for women, while life expectancy for highly educated adults is 82.6 years for men and 86.9 years for women [30]. This boils down to a difference of 6 to 7 years. The mere fact that the low-educated participants in our study, with a median age of 87, were still alive, mentally competent and able and willing to engage in this research indicates that their health situation and everyday functioning was better than that of the majority of their lowly educated peers.

Due to healthcare reforms in the Netherlands, admission policies for residential care homes have become more restrictive. Adults who previously would have been admitted to residential care homes now have to remain community-dwelling, while receiving care at home. This results in an increasing number of community-dwelling frail older adults. For these healthcare reforms to be successful, frail older adults at least partly need to manage their lives, health and healthcare by themselves [6]. However, the low levels of patient activation as found in our study indicate that the majority of frail older adults may not have the abilities, knowledge, skills and confidence to adequately engage to this level of self-management. This may have several consequences for their health and healthcare.

First of all, many older adults have multiple chronic conditions, often associated with disabilities, poor functional status and poor quality of life [31]. Their health and healthcare is further compromised if they also have low activation levels, as shown by Hibbard et al. [15].

A second possible consequence of older adults’ limited self-management skills is a higher number of hospital (re-)admissions and an increasing use of complex emergency care. As shown by Hibbard et al., less activated adults had higher rates of hospitalisations and emergency department visits than higher activated adults [10]. An increasing use of complex emergency care has been already observed in the Netherlands [32]. According to employees of emergency departments and ambulance control rooms of two Dutch provinces, this is due to higher numbers of community dwelling frail older adults as a consequence of recent policy chances that aim to reduce institutional care [32]. Also, general practitioners from all over the country and the primary care branch association have reported to experience greater work burden due to more urgent care demands outside office hours from frail older adults, who now remain community-dwelling [33].

Third, limited self-management skills of older adults may result in a higher than expected need of (informal) caregiver assistance. Recent studies already demonstrated an increasing need for informal caregiver assistance [34]. This has been associated with high levels of caregiver burden and several health-related problems, such as sleep-disturbances and depressive symptoms [35, 36].

Previous studies have shown that patient activation can be improved by e.g. clinical-based or community-based interventions, which allow adults to be supported in the development of their self-management skills [10, 11]. However, as these studies were performed in young and middle-aged adults [10, 11], it is unknown whether patient activation is still modifiable in frail, older adults. It has been argued that efforts to promote patient activation are ethically justified because of two reasons. First, the right to self-determination of adults will be addressed by allowing them to set health goals and by promoting their ability to accomplish these goals [37]. The second justification is a consequentialist one: evidence shows that efforts to promote patient activation are likely to produce better health and health care outcomes [37]. However, expecting adults to actively promote their own health and healthcare is only justified when they have the capacity to do so and when others create a realistic opportunity for them to do so [37], e.g. by delivering healthcare tailored to their care needs and activation levels.

This study has several strengths. We conducted personal interviews assuring that participants understood the questions correctly and that we interpreted their answers appropriately. Furthermore, we were able to include study participants with the exceptionally high median age of 87 years. There are some limitations, which should be considered when interpreting the findings. The response rate of our study was modest. Therefore, our findings are not necessarily generalisable to all frail, older adults who receive care. Furthermore, our study population consisted of mentally competent older adults who were able and willing to engage in research and who were potentially more interested in conversations about health and healthcare than decliners. This may have resulted in an overestimation of the level of patient activation in this population. On the other hand, the TFI may be more likely to identify people who have lower levels of activation. Finally, this study had a cross-sectional design. We were not able to draw conclusions concerning the direction of associations between health characteristics and patient activation.

## Conclusions

The majority of this frail and very old study-population, especially those with a lower health-related quality of life, may be unable to manage their health and healthcare to the level expected of them. The increasing population of frail older adults may need help in managing their health and healthcare.
